# iFCR方案一线治疗慢性淋巴细胞白血病/小淋巴细胞淋巴瘤长期随访结果及MRD在其中的应用价值

**DOI:** 10.3760/cma.j.cn121090-20251125-00549

**Published:** 2026-04

**Authors:** 铭 刘, 业钦 沙, 姝超 秦, 奕 夏, 祎 缪, 翰宁 唐, 雨洁 吴, 纯 乔, 磊 范, 建勇 李, 华渊 朱

**Affiliations:** 1 南京医科大学第一附属医院，江苏省人民医院血液科，南京 210029 Department of Hematology, The First Affiliated Hospital of Nanjing Medical University, Jiangsu Province Hospital, Nanjing 210029, China; 2 南京医科大学附属宿迁第一人民医院，江苏省人民医院宿迁医院血液科，宿迁 223999 Department of Hematology, The Affiliate Suqian First People's Hospital of Nanjing Medical University, Suqian Branch of Jiangsu Provincial People's Hospital, Suqian 223999, China

**Keywords:** 白血病，淋巴细胞，慢性，B细胞, 小淋巴细胞淋巴瘤, 可检测残留病, Leukemia, lymphocytic, chronic, B-cell, Small lymphocytic lymphoma, Measurable residual disease

## Abstract

**目的:**

探索可检测残留病（MRD）在iFCR（伊布替尼联合氟达拉滨、环磷酰胺、利妥昔单抗）方案治疗慢性淋巴细胞白血病/小淋巴细胞淋巴瘤（CLL/SLL）中的临床价值。

**方法:**

回顾性收集并分析2019年2月至2025年6月在南京医科大学第一附属医院血液科接受iFCR方案治疗的34例初治CLL/SLL患者的治疗过程及随访阶段以流式细胞术（FCM）及二代测序（NGS）检测的MRD数据，并与疗效评估、无进展生存（PFS）、总生存（OS）等指标综合分析。uMRD4定义为经FCM检测MRD<10^−4^。根据外周血MRD（PB-MRD）动力学将患者分为持续uMRD4组（13例）、MRD复阳组（4例）和MRD持续阳性组（7例）。

**结果:**

中位随访时间60.2（95％ *CI*：53.9～66.5）个月，患者总体中位PFS期和OS期均未达到。5年PFS率和OS率分别为58.6％（95％ *CI*：35.6％～96.6％）和94.4％（95％ *CI*：84.4％～100％）。与免疫球蛋白重链可变区（IGHV）无突变和TP53异常的患者相比，IGHV突变（中位PFS期：未达到对59.3个月，*P*＝0.019）和TP53无异常（中位PFS期：未达到对38.6个月，*P*＝0.011）的患者PFS期更长。联合治疗结束2个月后达PB-uMRD4患者与PB-MRD阳性患者相比获得完全缓解（CR）率更高（73.3％对20.0％，*P*＝0.015），且长期随访中PB-uMRD4持续时间更长［（36.3±16.2）个月对（4.6±4.7）个月，*P*<0.001］。经FCM（*P*<0.001，*r*＝0.739）和NGS（*P*<0.001，*r*＝0.869）检测的PB-MRD与骨髓MRD（BM-MRD）具有较好的一致性，而在同一样本中，FCM和NGS检测PB-MRD的一致性（*P*＝0.003，*r*＝0.439）优于BM-MRD（*P*＝0.033，*r*＝0.299）。患者接受iFCR方案治疗至2年时根据BM-MRD水平选择主动停药（达uMRD4者，9例）或继续维持（MRD阳性，19例），至末次随访时分别有1例（1/9，11.1％）和7例（7/19，36.8％）患者出现疾病进展，而其中7例主动停药保持PB-uMRD4且CR状态者均不合并del（17p）、del（11q）和TP53突变。持续uMRD4组较MRD持续阳性组复杂核型（7.7％对57.1％，*P*＝0.031）、del（17p）（0对42.9％，*P*＝0.031）、TP53突变（0对42.9％，*P*＝0.031）患者比例低。

**结论:**

IGHV无突变及TP53异常是接受iFCR方案一线治疗的CLL/SLL患者PFS的不利因素。联合治疗结束2个月后达PB-uMRD4患者有望保持长时间PB-uMRD4，而伴有复杂核型、del（17p）、TP53突变等高危因素者达到uMRD并停药的可能性小；根据治疗2年内的BM-MRD水平调整伊布替尼维持策略可行，动态监测PB-MRD有助于发现MRD复阳和疾病进展。

## Abstract (en)

**Objective:**

This study aimed to investigate the clinical significance of measurable residual disease（MRD）in patients with chronic lymphocytic leukemia/small lymphocytic lymphoma（CLL/SLL）treated with ibrutinib plus fludarabine, cyclophosphamide, and rituximab（iFCR）regimen.

**Methods:**

Data of 34 patients with treatment-naive CLL/SLL treated with the iFCR regimen in the Department of Hematology of The First Affiliated Hospital of Nanjing Medical University from February 2019 to June 2025 were retrospectively collected and comprehensively analyzed. Data included MRD detected with flow cytometry（FCM）and NGS, efficacy evaluation, progression-free survival（PFS）, and overall survival（OS）. UMRD4 was defined as MRD of <10^−4^ detected with FCM. According to peripheral blood MRD（PB-MRD）kinetics, patients were categorized into three groups: sustained uMRD4（*n*＝13）, MRD relapse（*n*＝4）, and sustained MRD positive（*n*＝7）.

**Results:**

Median follow-up was 60.2（95％ *CI*: 53.9–66.5）months, and the median PFS and median OS times were not reached. The 5-year PFS and OS rates were 58.6％（95％ *CI*: 35.6％–96.6％）and 94.4％（95％ *CI*: 84.4％–100％）, respectively. Compared with patients with unmutated immunoglobulin heavy chain variable（IGHV）and TP53 aberration, patients demonstrating mutated IGHV（median PFS time: not reached *vs* 59.3 months, *P*＝ 0.019）and the absence of TP53 aberration（median PFS time: not reached *vs* 38.6 months, *P*＝0.011）were associated with a longer PFS. After 2 months of combination therapy completion, patients with PB-uMRD4 achieved a higher rate of complete response（CR）compared with those with positive PB-MRD（73.3％ *vs* 20.0％, *P*＝0.015) and demonstrated a longer PB-uMRD4 duration during long-term follow-up（36.3±16.2 months *vs* 4.6±4.7 months, *P*<0.001）. A good concordance was observed between PB-MRD and bone marrow MRD（BM-MRD）detected with FCM（*P*<0.001, *r*＝0.739）and NGS（*P*<0.001, *r*＝0.869）. Further, the concordance of PB-MRD detection（*P*＝0.003, *r*＝0.439）between FCM and NGS was superior to that of BM-MRD（*P*＝0.033, *r*＝0.299）. Treatment was either actively discontinued（those achieving uMRD4）or continued（those with detectable MRD）according to the BM-MRD level after 2 years of iFCR treatment. Furthermore, disease progression occurred in 1（1/9, 11.1％）patient who discontinued therapy and 7（7/19, 36.8％）patients who continued treatment. Among them, 7 patients who actively discontinued treatment and kept PB-uMRD4 and CR demonstrated no del（17p）, del（11q）, and TP53 mutations. The proportion of patients with complex karyotypes（7.7％ *vs* 57.1％, *P*＝0.031）, del（17p）（0 *vs* 42.9％, *P*＝0.031）, and TP53 mutations（0 *vs* 42.9％, *P*＝0.031）was lower in the sustained uMRD4 group compared with the sustained MRD-positive group.

**Conclusion:**

Unmutated IGHV and TP53 aberration remained adverse factors for PFS in patients with CLL/SLL who received the first-line iFCR regimen. After 2 months of combination therapy completion, patients with PB-uMRD4 were more likely to maintain PB-uMRD4 for an extended period, whereas patients with high-risk factors, including complex karyotype, del（17p）, and TP53 mutation, were less likely to achieve uMRD and discontinue treatment. Furthermore, the ibrutinib maintenance strategy can be adjusted based on the BM-MRD level at 2 years. Monitoring PB-MRD dynamically was crucial in detecting MRD conversion and progression.

慢性淋巴细胞白血病（CLL）是一种成熟B淋巴细胞克隆增殖性肿瘤，可累及外周血（PB）、骨髓（BM）、淋巴结及脾，需根据患者分子生物学特征及体能状态进行分层治疗[1]。FCR方案（氟达拉滨+环磷酰胺+利妥昔单抗）曾作为年轻和体能状态良好的CLL/小淋巴细胞淋巴瘤（CLL/SLL）患者一线治疗方案，Tam等[2]研究的长期随访显示FCR方案完全缓解（CR）率为72％，6年总生存（OS）率和无进展生存（PFS）率分别为77％和51％，免疫球蛋白重链可变区（IGHV）突变的患者具有更长的PFS期和OS期。布鲁顿酪氨酸激酶抑制剂（BTKi）的出现改变了CLL/SLL的治疗模式，但长期单药维持存在缓解深度不足、不良反应管理及经济负担等问题[3]–[4]。因此，既往研究探索了BTKi联合FC（氟达拉滨+环磷酰胺）及抗CD20单抗的治疗方案，将可检测残留病（MRD）纳入评估指标并根据MRD调整疗程及维持治疗时间，研究结果显示该种治疗模式能在部分患者中达到更深的缓解、更高的MRD低于检测阈值（uMRD）率[5]–[6]，提示MRD具有替代PFS作为预后指标并指导治疗的价值[7]。本中心曾开展一项单臂、真实世界研究，探索了伊布替尼联合FCR（iFCR）方案在一线CLL/SLL中的疗效与安全性[8]。本研究对34例接受iFCR方案治疗的患者的MRD及生存进行长期随访，分析不同检测方法及样本来源的MRD一致性及影响达到uMRD及PB-MRD动力学的因素，探索及验证MRD在以iFCR方案为代表的新药联合治疗方案中评估停药、预测复发、调整治疗方案等临床实践的价值。

## 病例与方法

1. 病例：本研究为回顾性队列研究，纳入2019年2月至2025年6月间在南京医科大学第一附属医院血液科接受iFCR方案治疗的初治CLL/SLL患者34例。所有患者均符合iwCLL 2018年指南[9]诊断标准并达到疾病治疗指征。纳入患者达治疗指征时的临床资料，包括性别、年龄、疾病分期、B症状、血常规、生化检查、影像学结果、MRD结果、IGHV突变状态、CpG刺激染色体核型分析、FISH结果、CLL相关致病基因二代测序（NGS）检测结果等。启动治疗时患者总体中位年龄为55（48，57）岁，其中CLL 32例、SLL 2例。CLL患者Rai分期Ⅲ～Ⅳ期占62.5％（20/32），Binet分期C期占59.4％（19/32）。患者总体IGHV无突变61.8％（21/34），TP53突变17.6％（6/34），TP53突变的6例患者中有5例同时伴有del（17p），复杂核型（CK）32.4％（11/34）。本研究已经南京浦口区中心医院伦理委员会批准（批件号：2021-SR-006）。

2. 治疗方案：iFCR方案治疗的第1个周期所有患者均接受伊布替尼治疗（420 mg/d）共7 d，后联合FCR方案（利妥昔单抗375 mg/m^2^，第0天；氟达拉滨25 mg/m^2^，第1～3天；环磷酰胺250 mg/m^2^，第1～3天）。28 d为1个周期，根据患者疗效及耐受性至多6个周期，随后以伊布替尼（420 mg/d）维持治疗2年后依据iwCLL标准评估患者达到CR或CR伴骨髓不完全恢复（CR/CRi）且经流式细胞术（FCM）检测BM-MRD低于检测阈值（10^−4^为阈值，BM-uMRD4）可停药随访[8]，否则继续接受伊布替尼单药治疗直至疾病进展或无法耐受不良反应。

3. MRD检测方法及相关定义：MRD检测方法包括FCM及NGS，FCM按照本中心实验室常规方法[10]；NGS检测按照先声医学诊断实验方法[11]。uMRD定义：每10 000个白细胞中CLL细胞<1个（MRD<10^−4^）[9]。本研究定义uMRD4为经FCM检测MRD<10^−4^；uMRD6为经NGS检测MRD<10^−6^。MRD复阳定义[12]：MRD<10^−4^后，在3～6个月内连续2次MRD≥10^−4^。PB-uMRD4持续时间定义：在治疗中任何时间首次PB-MRD<10^−4^至末次检测PB-MRD<10^−4^的时间。首次达PB-uMRD4时间定义：自启动治疗至首次PB-MRD<10^−4^的时间。FCM检测MRD结果之间及FCM与NGS检测MRD结果之间以10^−4^为阈值，NGS检测MRD结果之间以10^−6^为阈值，在阈值同一侧的结果认为具有一致性。阳性预测值及阴性预测值以FCM-MRD结果及BM-MRD结果为参考。

4. 疗效与不良事件评估：CLL疗效评估参考iwCLL 2018标准[9]，SLL疗效评估参考Lugano 2014标准。联合治疗期间每个周期治疗前检测PB-MRD，第4个周期治疗前及联合治疗结束2个月后进行疗效评估，同时检测PB-MRD及BM-MRD。启动治疗后2年内每3至6个月，2年后每6个月至1年监测PB-MRD，部分有条件的患者同时通过NGS监测MRD。不良事件（AE）参考CTCAE 5.0。

5. 随访：通过门诊、住院病历及电话进行随访，末次随访时间为2025年6月1日。OS期定义为启动治疗至任何原因导致的死亡的时间。PFS期定义为启动治疗至首次出现疾病进展/复发或任何原因导致的死亡的时间。

6. 统计学处理：数据分析采用SPSS 25、Graphpad Prism（10.1.2版本）软件。计数资料采用例数（百分数）描述，计量资料采用*x*±*s*描述或*M*（*Q*_1_，*Q*_3_）。采用Kaplan-Meier法绘制生存曲线并使用Log-rank检验进行差异分析。中位随访时间采用反Kaplan-Meier法进行估计。*P*<0.05认为差异有统计学意义。

## 结果

1. 治疗过程、疗效及不良事件：34例患者在接受7 d的伊布替尼单药导入期后接受iFCR方案治疗。其中23例患者接受了3～4个周期iFCR方案治疗，9例接受了6个周期iFCR方案治疗，1例患者接受2个周期iFCR方案治疗后失访，1例患者接受2个周期iFCR方案及4个周期伊布替尼治疗后选择维奈克拉治疗。患者总体第4个周期治疗前CR/CRi率、PB-uMRD4率、BM-uMRD4率和CR/CRi且BM-uMRD4率分别为35.3％（12/34）、55.9％（19/34）、41.2％（14/34）和17.6％（6/34），至联合治疗结束2个月后分别提升至55.9％（19/34）、64.7％（22/34）、55.9％（19/34）和38.2％（13/34）。

31例患者在联合治疗结束后接受伊布替尼维持治疗，期间有1例患者因疾病进展停止维持治疗，1例因失访停止维持治疗，1例因AE停止维持治疗。治疗满2年达到停药标准9例（32.1％，9/28），停药后中位随访时间为39.8（95％ *CI*：37.9～41.6）个月，其中7例截至末次随访仍保持PB-uMRD4且CR状态，IGHV突变57.1％（4/7），CK 28.6％（2/7），无del（17p）、del（11q）和TP53突变。19例（67.9％，19/28）BM-MRD阳性患者继续口服伊布替尼，IGHV无突变63.2％（12/19），CK 33.3％（6/18），del（17p）15.8％（3/19），TP53突变21.1％（4/19）；其中14例（73.7％，14/19）BM-MRD及PB-MRD阳性的患者维持治疗中4例（28.6％，4/14）PB-MRD转阴；5例（35.7％，5/14）至末次检测始终PB-MRD阳性［中位随访时间60.2（95％ *CI*：27.2～93.2）个月］，IGHV无突变60.0％（3/5），CK 50.0％（2/4），TP53突变20.0％（1/5），无del（17p），其中3例（60.0％，3/5）分别在随访32.0、33.3、38.7个月时自行选择联合维奈克拉治疗。7例（36.8％，7/19）口服伊布替尼的患者出现临床进展，其中IGHV无突变85.7％（6/7），同时合并CK、del（17p）、TP53突变42.9％（3/7）；7例（36.8％，7/19）最终达PB-uMRD4，中位伊布替尼单药维持时间为53.2（95％ *CI*：51.4～55.0）个月，其中IGHV无突变42.9％（3/7），CK 14.3％（1/7），无del（17p）及TP53突变。

在iFCR方案治疗及伊布替尼维持治疗期间，所有患者均至少发生1次AE。最常见的血液学AE为中性粒细胞计数降低（25/34，73.5％），其中3～4级占67.6％（23/34），其次为PLT降低（24/34，70.6％），其中3～4级占35.3％（12/34），而贫血发生率较低，占58.8％（20/34），其中3～4级占14.7％（5/34）。常见的非血液学AE包括恶心（21/34，61.8％）、全身乏力（16/34，47.1％）、皮疹（16/34，47.1％）。8例患者发生3级的感染事件，其中6例为肺部感染。伊布替尼维持治疗期间，17.6％（6/34）患者发生贫血，均为1～2级；44.1％（15/34）患者发生中性粒细胞计数降低，其中3～4级占35.3％（12/34）；61.8％（21/34）发生PLT降低，其中3～4级占17.6％（6/34）；20.6％（7/34）患者发生感染，其中3～4级占11.8％（4/34）。

2. 生存分析：患者总体中位随访时间为60.2（95％ *CI*：53.9～66.5）个月，中位PFS期和OS期均未达到。3年PFS率和OS率分别为83.1％（95％ *CI*：70.6％～97.8％）和100％（95％ *CI*：100％～100％），5年PFS率和OS率分别为58.6％（95％ *CI*：35.6％～96.6％）和94.4％（95％ *CI*：84.4％～100％）。9例患者进展，中位进展时间为31.7（95％ *CI*：30.2～33.3）个月，其中1例接受6个周期iFCR方案治疗，并以伊布替尼维持治疗3.1个月后诊断转化为弥漫大B细胞淋巴瘤（DLBCL）。根据MRD分组进行生存分析比较，结果差异虽无统计学意义，但联合治疗结束2个月后达CR且PB-uMRD4（11例）与达部分缓解（PR）且PB-MRD阳性（8例）的患者相比显示出更长的PFS期趋势（中位PFS期：68.8个月对52.6个月，*P*＝0.207），在达CR且BM-uMRD4（8例）与达PR且BM-MRD阳性（6例）的患者之间展现出同样的趋势（中位PFS期：68.8个月对45.9个月，*P*＝0.121）。合并IGHV无突变［无突变（21例）对突变（13例），中位PFS期：59.3个月对未达到，*P*＝0.019］、TP53异常［异常（包括突变与缺失，6例）对无异常（28例），中位PFS期：38.6个月对未达到，*P*＝0.011］的患者PFS期更短。而合并CK（11例）与无CK（22例）患者PFS差异无统计学意义（中位PFS期：未达到对未达到，*P*＝0.191）。

3. MRD一致性分析：本研究探索了在此治疗模式下不同检测方法及样本来源的MRD一致性（图1）。本研究收集了同一时间点经FCM检测PB-MRD和BM-MRD共119对样本，其中96对（80.7％）MRD结果一致，23对（19.3％）不一致。不一致的样本中21对为PB-uMRD4而BM-MRD阳性，PB-MRD的阳性预测值为95.1％（39/41），阴性预测值为73.1％（57/78）。同一时间点经NGS检测PB-MRD和BM-MRD共34对样本，其中32对（94.1％）MRD结果一致，2对（5.9％）不一致，PB-MRD的阳性预测值为95.7％（22/23），阴性预测值为90.9％（10/11）。

**图1:**
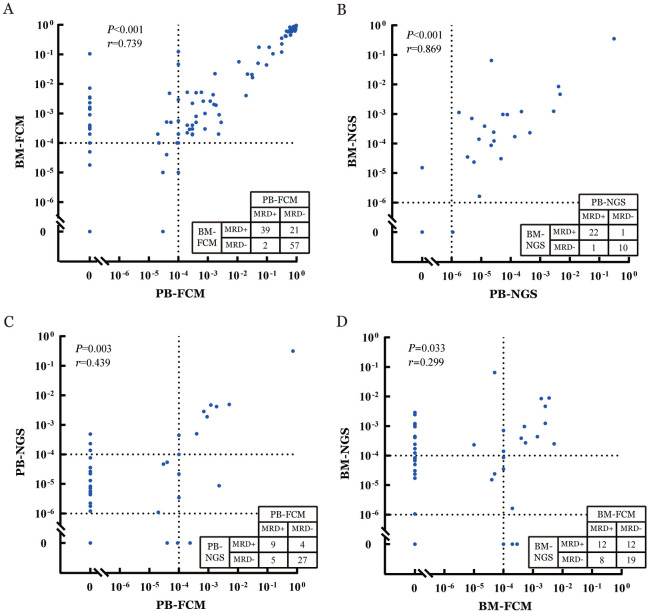
慢性淋巴细胞白血病/小淋巴细胞淋巴瘤患者MRD一致性分析 **A** 同一时间点经FCM检测PB-MRD和BM-MRD共119对；**B** 同一时间点经NGS检测PB-MRD和BM-MRD共34对；**C** 同一时间点经FCM与NGS检测PB-MRD结果共45对；**D** 同一时间点经FCM与NGS检测BM-MRD结果共51对 **注** MRD：可检测残留病；BM：骨髓；PB：外周血；FCM：流式细胞术；NGS：二代测序

同一时间点经FCM与NGS检测PB-MRD结果共45对，其中36对（80.0％）MRD结果一致，9对（20.0％）不一致，其中4对FCM检测为uMRD4而NGS检测为阳性，NGS-MRD的阳性预测值为69.2％（9/13），阴性预测值为84.3％（27/32）。同一时间点经FCM与NGS检测BM-MRD结果共51对，其中31对（60.8％）MRD结果一致，20对（39.2％）不一致。不一致的样本中12对FCM检测为uMRD4而NGS检测为阳性，NGS-MRD的阳性预测值为50.0％（12/24），阴性预测值为70.4％（19/27）。相关性分析结果示，用同一种方法检测PB和BM样本的MRD，FCM检测（*P*<0.001，*r*＝0.739）和NGS检测（*P*<0.001，*r*＝0.869）的一致性均较好；而在同一样本中，尽管用FCM与NGS两种方法的一致性仍较好，但PB一致性（*P*＝0.003，*r*＝0.439）优于BM（*P*＝0.033，*r*＝0.299）。

4. PB-MRD动力学及其影响因素和MRD影响因素分析：采用FCM对24例患者进行PB-MRD随访，根据患者启动治疗2年内的PB-MRD变化特点，将其分为3组（表1）即持续uMRD4组（13例），为在治疗中任何时间首次PB-MRD<10^−4^后并且在维持治疗期间保持MRD<10^−4^；MRD复阳组（4例）；MRD持续阳性组（7例），为PB-MRD在治疗中始终未低于检测阈值。另有10例患者因MRD检测不可及或表现为局部淋巴结进展暂不归入上述分组。持续uMRD4组CK（7.7％对57.1％，*P*＝0.031）、del（17p）（0对42.9％，*P*＝0.031）、TP53突变（0对42.9％，*P*＝0.031）患者比例低于MRD持续阳性组。进一步计算患者的PB-uMRD4持续时间和首次达PB-uMRD4时间并分析其影响因素，IGHV突变［IGHV突变对IGHV无突变：（39.8±18.8）个月对（22.4±16.4）个月，*P*＝0.031］、联合治疗结束2个月后达PB-uMRD4［PB-uMRD4对PB-MRD阳性：（36.3±16.2）个月对（4.6±4.7）个月，*P*<0.001］及BM-uMRD4［BM-uMRD4对BM-MRD阳性：（34.6±15.2）个月对（12.8±16.5）个月，*P*＝0.010）］的患者PB-uMRD4持续时间更长，而出现del（11q）［（16.9±8.6）个月对（32.5±20.0）个月，*P*＝0.017］的患者PB-uMRD4持续时间更短。早期评估节点（第4个周期治疗前）的PB-MRD及BM-MRD状态对于PB-uMRD4持续时间的影响差异均无统计学意义（*P*值均>0.05）。ATM无突变的患者首次达PB-uMRD4时间更短（*P*＝0.031），中位首次达PB-uMRD4时间为2.8（95％ *CI*：1.9～3.7）个月；在联合治疗结束2个月后PB-uMRD4患者与PB-MRD阳性患者相比获得CR率更高（73.3％对20.0％，*P*＝0.015）。

**表1:** 不同PB-MRD动力学特征的患者分子生物学特征比较［例数/总例数（％）］

分子生物学特征	持续uMRD4组（13例）	MRD复阳组（4例）	MRD持续阳性组（7例）	*P*1值	*P*2值	*P*3值
IGHV无突变	7/13（46.2）	2/4（50.0）	5/7（71.4）	>0.05	0.642	0.576
CK	1/13（7.7）	0/3（0）	4/7（57.1）	>0.05	0.031	0.200
del（17p）	0/13（0）	0/4（0）	3/7（42.9）	–	0.031	0.236
del（13q）	7/12（58.3）	1/4（25.0）	6/7（85.7）	0.569	0.333	0.088
del（11q）	1/13（7.7）	0/4（0）	2/7（28.6）	>0.05	0.270	0.491
Cen 12	1/11（9.1）	1/3（33.3）	1/6（16.7）	0.396	>0.05	>0.05
TP53突变	0/13（0）	1/4（25.0）	3/7（42.9）	0.235	0.031	>0.05
NOTCH1突变	1/13（7.7）	2/4（50.0）	0/7（0）	0.121	>0.05	0.109
SF3B1突变	0/13（0）	1/4（25.0）	1/7（14.3）	0.235	0.350	>0.05
ATM突变	2/13（15.4）	1/4（25.0）	1/7（14.3）	>0.05	>0.05	>0.05
MYD88突变	2/13（15.4）	1/4（25.0）	0/7（0）	>0.05	0.521	0.364

**注** PB：外周血；MRD：可测量残留病；uMRD4：MRD<10^−4^；IGHV：免疫球蛋白重链可变区；CK：复杂核型；Cen 12：12号染色体三体；*P*1：持续uMRD4组与MRD复阳组比较；*P*2：持续uMRD4组与MRD持续阳性组比较；*P*3：MRD复阳组与MRD持续阳性组比较

## 讨论

MRD是一种反映肿瘤负荷的敏感指标，可作为疗效评估的补充并预测PFS，动态监测MRD可观察MRD清除速率、MRD复发情况，为指导治疗提供依据[13]。目前MRD的检测方法有多参数流式细胞术（MFC）、等位基因特异性聚合酶链反应（ASO-PCR）、NGS等，由于检测技术敏感性不同，得到的MRD结果可能存在差异[13]–[14]。既往研究比较了在多发性骨髓瘤中MFC与NGS的MRD结果一致性，以10^−5^为阈值，68.0％的MRD结果一致[15]。本研究对iFCR方案治疗下不同检测方法及样本来源的MRD进行了一致性分析，结果表明相同方法检测的PB-MRD与BM-MRD间具有较好的一致性，PB-MRD可预测BM-MRD，且NGS方法的预测能力更强。但在相同部位，NGS检测MRD的敏感性高于FCM，导致不一致的MRD结果较多，且在BM中更为明显，其原因可能是iFCR方案对PB肿瘤细胞的清除能力较BM更强。因此，我们推荐在有限期治疗探索中，可经FCM检测患者达PB-uMRD4后，再进一步检测BM-MRD，避免多次骨髓穿刺对患者造成经济及心理上的负担，提高患者的依从性及临床可行性。

从化学免疫治疗时代到伊布替尼、维奈克拉等靶向药物治疗时代，MRD逐步成为一种可靠的能够替代PFS结局指标[7]。在化学免疫治疗时代的一线治疗及多线治疗中，MRD可作为独立预后因素，低BM-MRD水平与延长的PFS期相关[16]。E1912研究比较了FCR方案与伊布替尼联合利妥昔单抗方案的疗效，伊布替尼联合利妥昔单抗组在12个月时达PB-MRD<10^−1^的患者较PB-MRD>10^−1^的患者PFS期更长[17]。CLL14研究比较了维奈克拉联合奥妥珠单抗组与苯丁酸氮芥联合奥妥珠单抗组的疗效，两组治疗结束时BM-MRD阳性患者的PFS期均更短[18]。GAIA-CLL13研究将15个月时PB-MRD与PFS共同作为主要观察终点，维奈克拉联合奥妥珠单抗组与维奈克拉、奥妥珠单抗联合伊布替尼组达PB-uMRD的患者比例明显高于化学免疫治疗组[19]。而本研究MRD对PFS的影响无统计学意义，可能与样本量较小相关。同时，既往部分研究者也探索了根据MRD动态调整CLL/SLL个体化治疗的策略。Strati等[20]的研究表明FCR方案治疗结束后BM-uMRD与更长的PFS期及OS期相关，接受3个周期FCR方案治疗后达uMRD停止治疗的患者与3个周期FCR方案治疗后达uMRD继续治疗的患者以及超过3个周期FCR治疗后达uMRD的患者相比，PFS差异无统计学意义，提示MRD快速清除的患者可减少疗程数。Thompson等[21]探索FCR方案治疗后MRD情况对于PFS的影响，并根据3个周期FCR方案治疗后BM-MRD水平对患者进行危险分层，指出MRD>1％的患者应调整为新型靶向药物治疗。Soumerai等[22]探索了以MRD为指导的BOVen方案治疗，89％的患者达到PB-uMRD和BM-uMRD且符合设定的停药标准并停药，中位治疗周期为10个周期，达到BM-uMRD的中位时间为8个月，其中快速达uMRD（ΔMRD400）的患者即使接受的药物暴露时间较短，仍存在PFS优势。上述以MRD为指导的治疗方案调整、复发预测表明监测MRD具有临床实践价值。

本队列既往结果表明CR/CRi率、PB-uMRD4率、BM-uMRD4率和CR/CRi且BM-uMRD4率在IGHV突变组（13例）与IGHV无突变且无TP53异常组间（15例）差异无统计学意义[8]。进一步分析了PB-MRD动力学及其影响因素。本研究数据提示伴有CK、del（17p）、TP53突变的患者更易出现MRD持续阳性，而IGHV突变、联合治疗结束2个月后达PB-uMRD4及联合治疗结束2个月后达BM-uMRD4的患者，PB-uMRD4持续时间更长。在7例MRD持续阳性患者中，4例患者选择联合维奈克拉治疗，治疗后2例达PB-uMRD4，另2例保持PB-MRD<10^−2^，但未发生临床进展；未选择联合维奈克拉的3例中，2例分别在随访31.2个月和45.9个月时发生临床进展。以上提示iFCR方案对于伴有上述3种高危因素的患者可能疗效不佳，可根据疗效评估选择联合维奈克拉治疗。而整体队列中长期随访结果提示IGHV无突变、TP53异常患者具有较差的PFS，支持上述的监测调整策略。长期随访显示，7例患者在停用伊布替尼后保持PB-uMRD4且CR状态，这7例无del（17p）和TP53突变，且IGHV突变及孤立性del（13q）分别占57.1％（4/7）及83.3％（5/6），其中4例患者送检联合治疗结束2个月后PB和BM-MRD且均低于检测阈值。对于缺少上述3种高危因素并在联合治疗结束2个月后达到PB及BM-uMRD4的IGHV突变患者有望长期保持PB-uMRD4并达停药标准停药。

本研究进一步探索影响MRD复阳的因素，但由于样本量较小未能发现具有统计学意义因素。在Niemann等[23]开展的I+V方案研究中，结果表明CK、TP53异常的患者更易出现MRD复阳（MRD>10^−2^）；而Ahn等[24]的iFCR研究中并未发现有统计学意义的PB-MRD动力学影响因素，其原因可能是样本量较小。本研究临床进展的9例患者中，2例规律随访的患者PB-MRD复阳早于临床进展，提示PB-MRD具有用于监测临床进展的价值；然而，随访中另外2例保持PB-uMRD4的CLL患者分别于随访27.2个月、31.7个月时进展，仅表现为淋巴结肿大并经穿刺证实转化为DLBCL，提示PB-MRD监测对于局部进展患者的局限性，而循环肿瘤DNA（ctDNA）可能会提供更多有效信息[25]。本中心既往研究随访结果显示在接受3个周期iFCR方案治疗后经NGS检测达到BM-uMRD6的3例患者持续保持CR/CRi且BM-uMRD6[8]，至末次随访仍保持PB-uMRD4，提示早期肿瘤细胞的快速清除可能预示着长期缓解，但仍需更大的样本量加以证实。因此，对于FCM检测达到PB-uMRD4及BM-uMRD4的患者，可考虑进一步行NGS评估MRD缓解深度，达到深度缓解的患者有更长的PFS期[26]。

本研究初步探索了MRD在iFCR方案治疗初治CLL/SLL中的临床价值，联合治疗结束2个月后的MRD可作为疗效的补充，对于治疗2年时患者能否停药具有预测价值，而在第4个周期前NGS检测达uMRD6的患者具有长期保持CR/CRi且uMRD的倾向。伴有CK、del（17p）、TP53突变等高危因素的患者在iFCR方案中达到uMRD且停药的可能性小，建议可开展BTKi联合BCL2抑制剂的有限周期探索。在长期随访中，以FCM动态检测PB-MRD以监测MRD复阳具有一定的临床可及性，出现MRD复阳的患者可增加随访次数以监测临床进展，而对于局部进展的患者仍需结合其他检查结果。本研究由于样本量较小，研究结论存在一定局限性，仍需要更大的样本量、更长的随访时间加以验证。
